# The evolution of enzyme function in the isomerases

**DOI:** 10.1016/j.sbi.2014.06.002

**Published:** 2014-06

**Authors:** Sergio Martinez Cuesta, Nicholas Furnham, Syed Asad Rahman, Ian Sillitoe, Janet M Thornton

**Affiliations:** 1 European Molecular Biology Laboratory, European Bioinformatics Institute EMBL-EBI, Wellcome Trust Genome Campus, Hinxton, Cambridge, CB10 1SD, United Kingdom; 2 Department of Pathogen Molecular Biology, London School of Hygiene & Tropical Medicine, Keppel Street, London, WC1E 7HT, United Kingdom; 3 Institute of Structural and Molecular Biology, Division of Biosciences, University College London, Gower Street, London, WC1E 6BT, United Kingdom

## Abstract

The advent of computational approaches to measure functional similarity between enzymes adds a new dimension to existing evolutionary studies based on sequence and structure. This paper reviews research efforts aiming to understand the evolution of enzyme function in superfamilies, presenting a novel strategy to provide an overview of the evolution of enzymes belonging to an individual EC class, using the isomerases as an exemplar.

## Highlights

• Isomerases usually evolve to become enzymes from other EC classes.
• Conservation of substrates is more common than conservation of bond changes.
• The relationship between functional similarity and sequence similarity is not linear.

**Current Opinion in Structural Biology** 2014, **26**:121–130

This review comes from a themed issue on **Sequences and topology**

Edited by **L Aravind** and **Christine A Orengo**

For a complete overview see the Issue and the Editorial

Available online 5th July 2014

**http://dx.doi.org/10.1016/j.sbi.2014.06.002**

0959-440X/© 2014 The Authors. Published by Elsevier Ltd. This is an open access article under the CC BY license (http://creativecommons.org/licenses/by/3.0/).

## Introduction

Enzymes are life's workforce. They catalyse the biochemical reactions that are the basis of metabolism in all living organisms. The major route for creating new enzyme functions is gene duplication and subsequent evolution of one enzyme to another with a novel, though usually related, function. Under the pressures of survival and reproduction, innovating new functions at the metabolic level allows organisms to adapt to an environment of changing chemical conditions [1]; for example, bacterial resistance to man-made chemicals such as drugs or pesticides.

### Previous work

Previous studies focusing on analysing enzyme superfamilies [2, 3] and directed evolution experiments [4] discovered aspects of how enzyme evolution is influenced by aspects of the chemistry of enzymes. The overall chemical reaction is often changed by recruiting different catalytic residues within an active site, whilst conserving a few residues required for the catalysis of at least one mechanistic step of the overall reaction [5]. Similarly, binding different substrates is commonly achieved by changing the residues involved in substrate binding and conserving residues involved in the overall reaction [6]. There is substantial evidence supporting changes of the overall chemical reaction [7], as well as results reporting the importance of binding different substrates in the evolution of function in superfamilies [8••, 9•, 10••]. Commonly, enzyme superfamilies evolve by a combination of these two strategies [11, 12]. For instance, phosphate binding sites are often conserved, whilst the rest of the substrate can be changed during evolution [13, 14].

Other comprehensive studies on the variation of enzyme sequence and structure [15, 16•] and plasticity of active sites [17, 18•] have also been fundamental in understanding how homologous enzymes accommodate alternative chemistries. Similarly, research on the convergent evolution of enzyme mechanisms [19] and active sites [20] presented nature's strategies to evolve different structural solutions for the catalysis of similar reactions [21, 22•, 23]. The widespread interest in understanding the evolution and chemistry of enzymes has led to large scale collaborative projects such as the Enzyme Function Initiative (EFI) [24] which aims to determine enzyme function using both experimental and computational approaches. Starting from a comprehensive alignment of genomic regions, Zhao and co-workers from the EFI have identified the epimerase activity, pathway context and biological role in osmoprotection of a structurally characterised enzyme of unknown function from *P. bermudensis* using a combination of virtual screening, metabolomics, transcriptomics and biochemical experiments [25^••^].

To explore this area further, we review our current knowledge of the evolution of the isomerase class of reactions, using newly developed computational tools to compare enzyme reactions [26^••^] and their evolution [27]. This is a specialised class of enzymes, which catalyse geometrical and structural rearrangements between isomers.

### Biological relevance of isomerases

**Figure 1:**
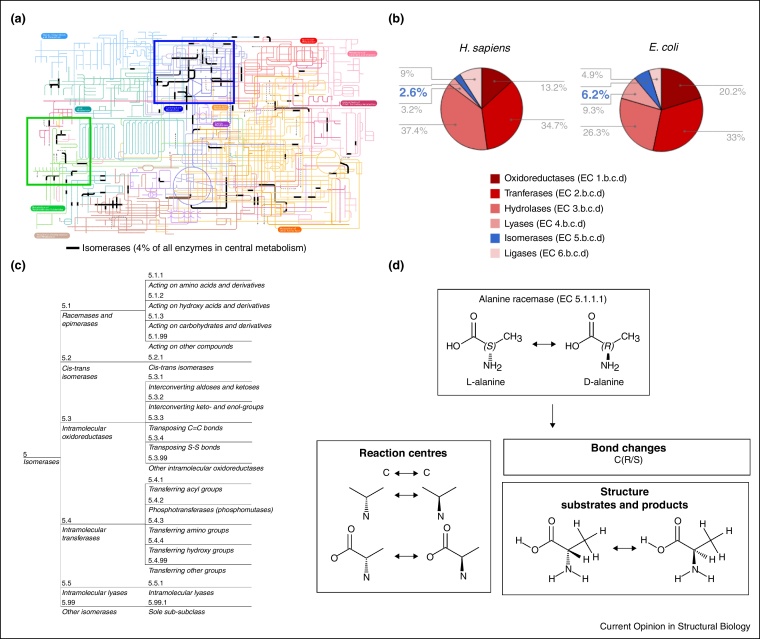
Biological importance of isomerases. **(a)** Core metabolic pathways (the isomerase reactions are emboldened in black). Carbohydrate and terpenoid/polyketide metabolic pathways are highlighted in blue and green squares, **(b)** Distribution of known enzymes in the human and *E. coli* genomes, **(c)** EC classification of isomerases. **(d)** Bond changes, reaction centres and structure of substrates and products obtained from the reaction catalysed by alanine racemase (EC 5.1.1.1) using EC-BLAST.

Isomerases are present in the metabolism and genome of most living organisms, catalysing up to 4% of the biochemical reactions present in central metabolism, in particular, carbohydrate metabolism. They also play a crucial role in the metabolism of terpenoids and polyketides that are important in generating secondary metabolites, especially in plants (Figure 1a).

The relative proportion of enzymes encoding for isomerase activity depends on the species. Whereas 2.6% of the genes encoding for enzymatic activity corresponds to isomerases in *Homo sapiens*, this proportion is higher in bacterial genomes such as *Escherichia coli* where they account for 6.2%. These figures correlate with the relative proportion of protein-coding genes encoding for enzymatic activity in general. Whereas in human, 20% of genes correspond to enzymes, this value increases to 37% in bacteria (Figure 1b).

The Nomenclature Committee of the International Union of Biochemistry and Molecular Biology (NC-IUBMB) maintains the most widely used functional classification of isomerases in the Enzyme Commission (EC) classification system [28]. Isomerases belong to the EC 5 primary class and they are grouped according to the chemistry of the reactions that they catalyse. They are subdivided in three hierarchical levels: 6 subclasses, 17 sub-subclasses and 231 serial numbers (Figure 1c). These serial numbers are associated with almost 300 biochemical reactions — for example EC 5.1.1.9 describes the racemisation of arginine, lysine or ornithine and it is therefore linked to three distinct reactions.

From a practical viewpoint, the total number of isomerase EC numbers (231) is small compared to other EC classes, which makes them attractive for manual analysis. Three of the six isomerase EC subclasses are similar to three EC primary classes (intramolecular oxidoreductases — EC 5.3 are designated from oxidoreductases — EC 1; intramolecular transferases — EC 5.4 from transferases — EC 2; and intramolecular lyases — EC 5.5 from lyases — EC 4, but refer to intramolecular reactions). Lastly, most of the isomerase reactions are unimolecular (one substrate and one product), which makes them relatively easy to compare.

Isomerases are used in many applications. In metabolic engineering, xylose isomerase (EC 5.3.1.5) has been traditionally used to convert glucose to fructose in the syrup industry and has recently been engineered to increase the yield of alcohol-based biofuels in *S. cerevisiae* [29]. In organic synthesis, several racemases and epimerases (EC 5.1) have been employed to resolve racemic mixtures in mild conditions and for the production of stereochemically pure amino acids [30]. Efforts in enzyme design have also managed to successfully convert racemases and epimerases acting on amino acids and derivatives (EC 5.1.1) into enzymes with lyase activity (EC 4) [31, 32]. Ultimately, some racemases and epimerases acting on amino acids are also targets for the development of antimicrobial drugs and the treatment of neuropathological disorders [33].

Studies linking chemical details of the catalytic reaction with how enzyme sequences evolve considering multiple enzyme superfamilies are scarce. Whereas some studies have focused on analysing only the chemistry [34, 35], other studies concentrated on sequence and structure evolution [15, 22•]. Some literature is however available addressing certain aspects of the chemistry and evolution of specific isomerases. In the 1990s, mandelate racemase (EC 5.1.2.2) and muconate-lactonizing enzyme (EC 5.5.1.1), members of the enolase superfamily, were among the first enzymes reported to be highly structurally similar yet catalysing different overall reactions. Several isomerases belonging to this superfamily have been studied over the last two decades [7]. Successive research efforts focused on ketosteroid isomerase (EC 5.3.3.1) have also been fundamental in understanding basic principles of enzyme catalysis [36^••^]. In addition, general strategies to assign isomerase specificity have been recently presented [37, 38, 25••], as well as comparative genomic techniques to discover new isomerases in bacterial genomes [39^•^]. Other investigations have partially explored isomerases in several superfamilies such as the haloacid dehalogenase, crotonase, vicinal oxygen chelate, amidohydrolase, alkaline phosphatase, cupin, short-chain dehydrogenase/reductase and PLP-dependent aspartate amino-transferase superfamilies [23, 40, 41, 42•, 43].

## Methods for analysing sequence, structure and functional relationships

Protein similarity networks have been used very successfully to map biological information to large sets of proteins [44, 43]. However, it is also necessary to include associated changes of catalytic function during evolution preferably in an automated fashion. FunTree is a resource developed to accomplish that goal [27] and it is maintained in collaboration with the CATH classification of protein structures [45]. By combining sequence, structure, phylogenetic, chemical and mechanistic information, it allows one to answer fundamental questions about the link between enzyme activities and their evolutionary history in the context of superfamilies. FunTree uses phylogenetic methods to infer ancestral enzymes in superfamilies and estimate their most likely functions [46]. By traversing the generated phylogenetic tree from ancestor to modern enzymes, explicit changes of function are identified between groups of enzymes belonging to a superfamily. Ultimately, each functional change is represented by two sets of enzymes catalysing two distinct functions so both functions and enzymes are comparatively analysed using functional and all-against-all sequence similarity.

To explore the evolution of the isomerases, we have calculated the functional similarity between enzymes using EC-BLAST [26^••^], a recently developed algorithm to automatically compare biochemical reactions. This approach introduces three measures of functional similarity — comparison of bond changes, reaction centres and structure similarity of substrate(s) and product(s) — derived from the biochemical reaction catalysed by any given enzyme (Figure 1d). Bond changes refer to cleavage, formation and order change of chemical bonds and changes in stereochemistry of atoms and bonds. Reaction centres are molecular substructures representing the local environment around the atoms involved in bond changes. Last, the complete two-dimensional structures of substrate(s) and product(s) are also considered in the comparisons. These three measures are then combined with mechanistic data from MACiE [47] and extensive literature searches in order to inform our analyses.

## Review of current status and availability of data on isomerase reactions and their sequences

**Figure 2:**
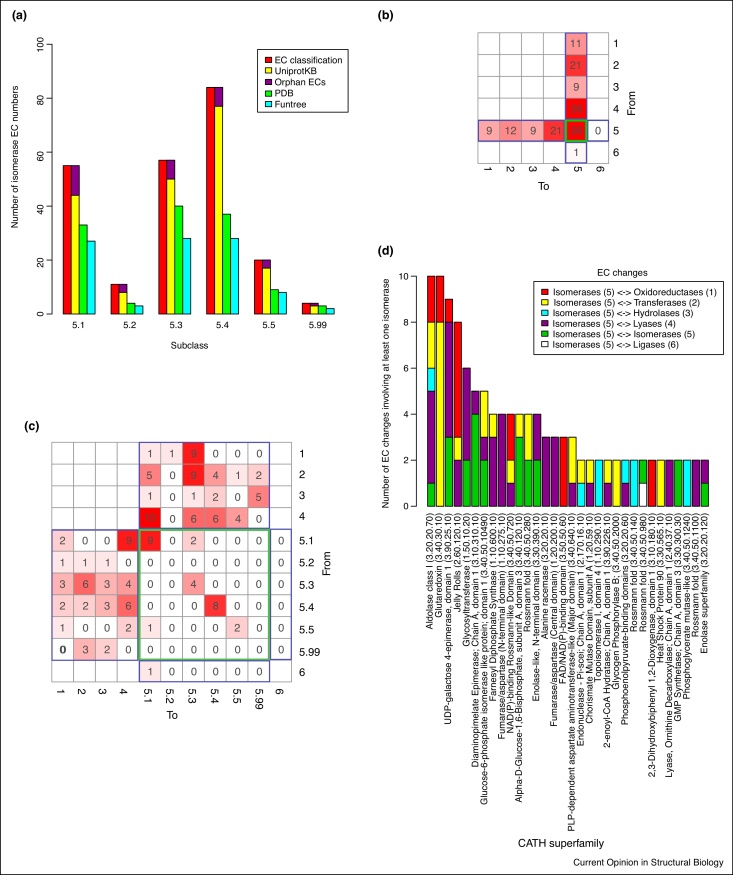
**(a)** Distribution of isomerases in EC classification, UniprotKB, PDB and FunTree. EC exchange matrices representing the changes in function during evolution of isomerases at the EC **(b)** class and **(c)** subclass levels. More frequent changes of isomerase function are highlighted in red. Green and blue boxes represent changes within isomerases and with other EC classes, respectively. **(d)** Frequency of EC changes involving isomerases by superfamily. The 32 superfamilies bearing multiple changes are illustrated.

Information related to the nomenclature of enzymes is publicly available in the ENZYME database [48]. It actively follows the recommendations of the NC-IUBMB and the 24-Jul-2013 version contained 231 current 4-digit isomerase EC numbers. 199 of them have sequence information in UniprotKB [49] and 32 are orphan isomerase EC numbers, also known as orphan enzymes [50, 51], a term given to EC numbers where no gene has been associated with these reactions and no sequence information is available in protein sequence repositories. Almost half of the isomerase EC numbers with sequence information (96) are present in FunTree [27] and Figure 2a shows the distribution by EC 5 subclass.

Protein structural data are available for 126 isomerase EC numbers, which have at least one entry in the PDB [52]. The 96 isomerases currently present in FunTree include domains, which are distributed across 81 CATH superfamilies: 17 are mostly alpha, 5 mostly beta and 59 mixed alpha/beta. Some superfamilies include more isomerases than others, for example, the superfamily UDP-galactose 4-epimerase, domain 1 (CATH 3.90.25.10) includes 7 racemases and epimerases (EC 5.1). In FunTree, one-third of the 96 isomerases including more than one domain superfamily (multidomain), with most of them including two or three superfamilies, but rarely more. Exceptionally, the subclass ‘other isomerases’ (EC 5.99), which has two EC numbers (EC 5.99.1.2 and 5.99.1.3) is distributed across seven and eight superfamilies, respectively. These are types I and II DNA topoisomerases, which are characterised by multiple domains required for the complex process of winding DNA [53].

## Observed changes of isomerase function

### Change in EC number

Analysis of FunTree data on 58 domain superfamilies identified a total of 145 unique changes of isomerase activity that occurred during evolution. Only one-fifth of the changes occur between isomerases whereas the rest involve changing from isomerases to perform reactions in other EC primary classes (Figure 2b). This is strikingly different from enzymes in other EC classes where changes in lower levels of the EC classification are more common than changes in the primary classification [8^••^]. Among the 26 changes within isomerases, only 3 change the EC subclass and 23 change the EC serial number, indicating a change in substrate (Figure 2c). A previous limited study of 24 pairs of enzymes reported that changes involving isomerases and lyases (EC 5↔EC 4) occur more often than changes to other EC classes [5]. Other analyses provided further evidence of these changes by revealing the structural insights of the evolution of an isomerase from a family of lyases, namely N-succinylamino acid racemase (EC 5.1.1.-) from o-succinylbenzoate synthases (EC 4.2.1.113) in the enolase superfamily [54]. Our comprehensive analysis confirms that such changes are indeed prevalent, with 39% of the 119 changes in primary classification involving lyases.

Most domain superfamilies show multiple changes of reaction chemistry involving different EC classes (Figure 2d). The most adaptable superfamily domains are aldolase class I (CATH 3.20.20.70) and glutaredoxin (CATH 3.40.30.10), each of them exhibiting 10 changes of isomerase function. Whereas the glutaredoxin ‘isomerase’ domain only exhibits changes of isomerase, oxidoreductase and transferase reactions, the aldolase class I domain has also evolved to become a hydrolase and lyase (Figure 2d).

### Correlation of sequence and function evolution

**Figure 3:**
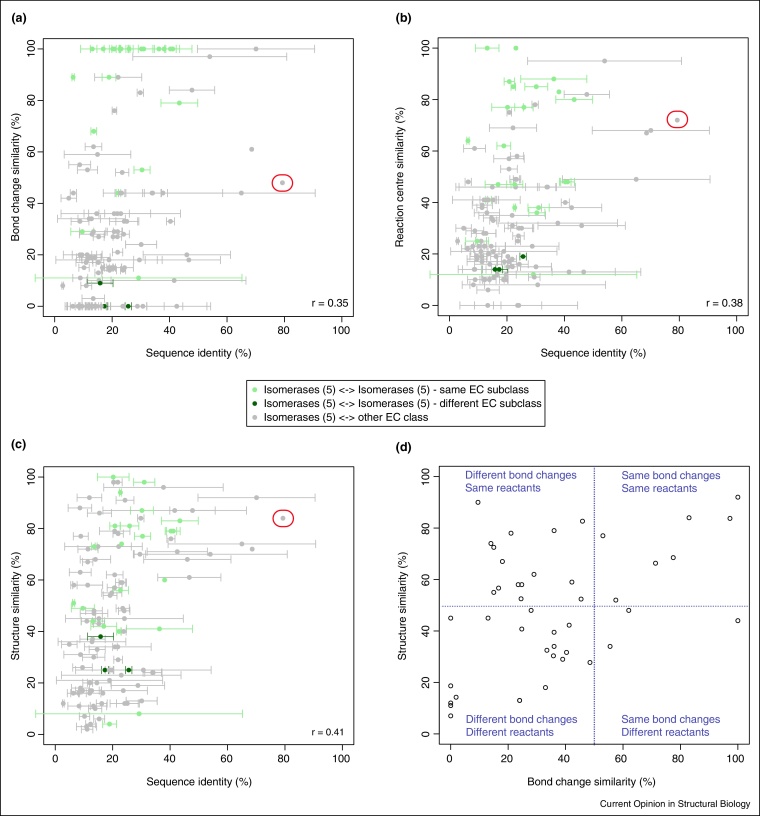
Sequence and functional similarity of the 145 changes of isomerase function. The three scatterplots represent global sequence identity against overall reaction similarity as calculated using three measures **(a)** bond change **(b)** reaction centre and **(c)** structure similarity of substrate(s) and product(s). Each point represents one change of enzyme function involving two sets of enzymes catalysing two distinct functions each [27]. Average global sequence identities and standard deviations (error bars) from all-against-all pairwise comparisons between sequences corresponding to one function and those corresponding to the second function. Circled in red, the change EC 4.2.1.124→EC 5.4.99.31 (see main text). Pearson's correlation coefficients (*r*) range from 0.35 to 0.41 and indicate weak but significant linear relationships (*p*-value < 0.001). **(d)** Distribution of bond change and structure similarities averaged by CATH superfamily.

To gain an overview of the relationship between sequence and functional divergence, an overall representation of the sequence and functional similarity between the homologous enzymes that perform different catalytic reactions is presented in Figure 3. This illustrates that most sequences have diverged considerably, with sequence identities in the range lower than 40%. The three measures of functional similarity (Figure 3a–c) capture different properties of the change in function, but none of the plots show any linear relationship between sequence and functional divergence. In addition, the distributions for each of these measures look quite different. In Figure 3a, which assesses the overall bond changes, there are two clusters, one consists of changes exhibiting bond change conservation when the isomerase EC subclass is maintained, and in the second changes at the isomerase EC subclass or EC primary class do not exhibit bond change conservation. This partition is not observed in the comparisons by reaction centres and structures of substrate(s) and product(s) and in overall, the similarities tend to be more uniformly spread (Figure 3b,c). Remarkably, there are only a few changes in which enzymes retain a relatively high degree of sequence and functional similarity. For instance, the glycosyltransferase superfamily (CATH 1.50.10.20) exhibits a change of arabidiol synthase (EC 4.2.1.124) into thalianol synthase (EC 5.4.99.31) (circled in red in Figure 3a–c). This change involves different enzyme sequences from the terpenoid biosynthesis pathway of *Arabidopsis thaliana* that share high sequence identity (79%) and high reaction similarity (48% — bond change, 72% — reaction centre and 84% — structure similarity). They both act on (S)-2,3-epoxysqualene as the main substrate to synthesise a different product, which explains why the structure similarity is high.

In an attempt to analyse the chemical diversity of the domain superfamilies performing changes of function in isomerases, we divided the functional similarity space in four quadrants as depicted in Figure 3d. Each point represents a superfamily whose changes of isomerase function were averaged according to overall chemistry — as measured by bond change similarity — and structures of the reactants — in line with the similarity of the structures of substrate(s) and product(s). Half of the superfamilies shared average similarities of reactants higher than 50% (top two quadrants), whereas only about one-fourth exhibited average similarities of overall chemistry higher than 50% (right two quadrants). Particularly, there are only three instances where the overall chemistry is similar but the structures of the reactants significantly diverge (bottom right quadrant), highlighting that this is a rare event in the evolution of isomerase function.

### An example — a family of SDRs acting on NDP-sugars from the UDP-galactose 4-epimerase superfamily

**Figure 4:**
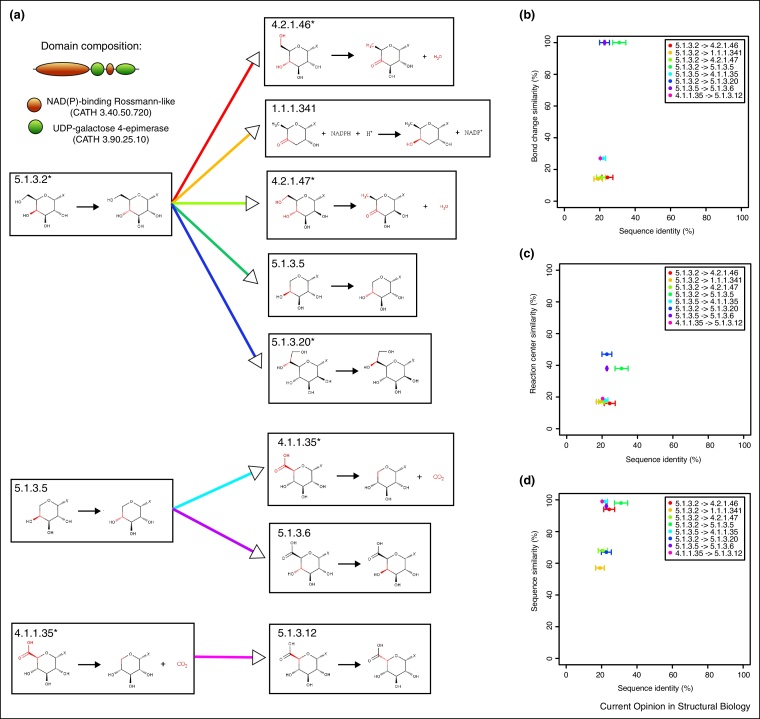
The evolution of SDRs acting on NDP-sugars. **(a)** Overview of the EC changes involving isomerases and domain composition of UDP-glucose 4-epimerases (EC 5.1.3.2). Biochemical reactions are represented in boxes. Black arrows inside boxes denote chemical transformations whereas coloured arrows linking boxes represent EC changes. EC numbers with an asterisk indicate reactions for which we found mechanistic evidence in MACiE [47] or in literature searches. Changing substructures are highlighted in red whereas X corresponds to nucleoside diphosphate moieties (ADP, TDP, GDP, CDP, UDP) in which the base may change, but the ribose diphosphate (or sometimes the 2′-deoxy derivatives) is broadly conserved. Three scatterplots illustrating sequence and functional similarity for this superfamily **(b)** bond change, **(c)** reaction centre and **(d)** structure similarity of substrate(s) and product(s) as in Figure 3.

To explore one set of changes in more detail we have studied eight changes of isomerase function involving a group of nine enzymes catalysing transformations between nucleoside diphosphate sugars (NDP-sugars). These metabolites are common in bacterial secondary metabolic pathways and they are necessary in molecular recognition and signalling processes [42^•^]. Several studies have revealed the structural, functional and mechanistic determinants of this group of evolutionary-related enzymes. They are epimerases (EC 5), dehydratases (EC 4), decarboxylases (EC 4) and oxidoreductases (EC 1) belonging to the subfamily of short-chain dehydrogenases/reductases (SDR) acting on NDP-sugars (Figure 4a) [55, 56, 57, 58]. The changes in function involve two-domain enzymes comprising a catalytic NAD(P)-binding Rossmann-like domain (CATH 3.40.50.720) and a domain known as UDP-galactose 4-epimerase (CATH 3.90.25.10), which confers substrate specificity. The active site is located in the interdomain cavity where a conserved Tyr, Lys and Ser/Thr form a catalytic triad. Reactivity takes place on the C4, C5 and C6 atoms of the sugar substructure through a mechanism involving a transient oxidation intermediate mediated by NAD [59]. The sequence data provide evidence that different catalytic amino acids are recruited to the active site in order to change the prevalent UDP-glucose 4-epimerase activity (EC 5.1.3.2) to other enzymatic activities. For instance, a base, Glu and an acid, Asp, are added to the catalytic triad in dTDP-glucose 4,6-dehydratase (EC 4.2.1.46) and GDP-mannose 4,6-dehydratase (EC 4.2.1.47) to perform the dehydration step which takes place in each of these overall reactions [58]. Since the reactivity takes place in the attached sugar moiety, the nucleoside diphosphate substructure (noted as X in Figure 4a) is not disrupted during catalysis and remains conserved in all enzymatic activities of this superfamily.

FunTree catalogues 8 changes of isomerase function within this family of enzymes (Figure 4a). They all share the same domain composition and therefore changes in function result directly from changes in sequence, rather than domain architecture. The analysis of sequence and functional similarities revealed that this family is divergent, with members sharing sequence identities in the 20–40% range. Bond change similarities revealed the already observed bimodal distribution due to the EC classification definitions (Figure 4b). Similarities by reaction centre remain low — not higher than 50% (Figure 4c) whereas overall, this set of functional changes tend to conserve structural similarity, due to the common binding of a conserved nucleoside diphosphate (Figure 4d).

Taken together, we think this overview of sequence and functional relationships may help identify possible sequences catalysing orphan isomerase EC numbers. For instance, comprehensive literature and database searches confirmed that the enzymatic activity UDP-glucosamine 4-epimerase (EC 5.1.3.16) is an orphan EC number. In 1959, it was first experimentally determined in rat liver by Maley [60]. The high functional similarity to the activities UDP-glucose 4-epimerase (EC 5.1.3.2), UDP-arabinose 4-epimerase (EC 5.1.3.5) and UDP-glucuronate 4-epimerase (EC 5.1.3.6) suggests that the sequence catalysing EC 5.1.3.16 may belong to the UDP-galactose 4-epimerase superfamily. Ultimately, experimental analysis will reveal whether candidate sequences actually perform this reaction.

## Conclusions

Using isomerases as an example, this review highlighted how enzyme chemistry may change over time, as enzymes evolve to perform different enzyme reactions.

Isomerases are a rare class of enzymes. Unlike other EC classes such as the ligases (EC 6), their functional classification is rather complex. While racemases, epimerases and cis-trans isomerases (EC 5.1 and 5.2) are sensibly grouped according to changes of stereochemistry, intramolecular oxidoreductases, intramolecular transferases and intramolecular lyases (EC 5.3, 5.4 and 5.5) are very similar to the chemistry of other EC classes. The subclass ‘other isomerases’ (EC 5.99) sits apart from other subclasses and exhibits great diversity, as evidenced by the distinct chemistry of DNA topoisomerases.

The surprising observation from our study highlights that isomerases are more likely to evolve new functions in different EC primary classes, rather than evolve to perform different isomerase reactions. This is unlike the other EC classes where more than two-thirds of the exchanges happen within the same EC class. In addition we note that exchanges between isomerases and lyases (EC 4) are prevalent.

Isomerases change their overall chemistry and conserve the structure of their substrates more often than conserving the chemistry and changing substrates. This is also unlike other types of enzymes and reflects the mechanisms of isomerases, which can often incorporate mechanistic components from different classes to provide a different overall outcome while conserving the substrate binding abilities.

This study is based on exploring the evolution of separate domains. However many enzymes are multidomain and change their domain composition and function during evolution [61]. Cataloguing the evolution of each one of the composite domains can lead to multiple different evolutionary pathways. Further analysis of multidomain architecture and more experimental data would complement and broaden this analysis.

The chaotic nature of the sequence and function relationship in superfamilies including isomerases is evidenced by the lack of correlation between sequence and functional similarity. Variations in sequence are always very large revealing that changes happened long ago, emphasizing that evolutionary studies need to be undertaken on a superfamily basis. Here we gave an example of how combining knowledge from the chemistry and evolution of enzymes acting on nucleoside diphosphate sugars may help to characterise related orphan activities.

## References and recommended reading

Papers of particular interest, published within the period of review, have been highlighted as:

• of special interest
•• of outstanding interest
